# Wide Carbon Nanopores as Efficient Sites for the Separation of SF_6_ from N_2_

**DOI:** 10.1038/srep11994

**Published:** 2015-07-07

**Authors:** Atsushi Takase, Hirofumi Kanoh, Tomonori Ohba

**Affiliations:** 1 Graduate School of Science, Chiba University, 1-33 Yayoi, Inage, Chiba 263-8522, Japan

## Abstract

SF_6_ and SF_6_-N_2_ mixed gases are used widely as insulators, but such gases have high greenhouse gas potential. The separation of SF_6_ from SF_6_-N_2_ mixed gases is an inevitable result of their use. Single-walled carbon nanohorns (CNHs) were used here for a fundamental study of the separation of SF_6_ and N_2_. The diameters of the interstitial and internal nanopores of the CNHs were 0.7 and 2.9 nm, respectively. The high selectivity of SF_6_ over N_2_ was observed only in the low-pressure regime in the interstitial 0.7 nm nanopores; the selectively was significantly decreased at higher pressures. In contrast, the high selectivity was maintained over the entire pressure range in the internal 2.9-nm nanopores. These results showed that the wide carbon nanopores were efficient for the separation of SF_6_ from the mixed gas.

SF_6_ is used as an insulator for high voltage breakers and circuit breakers in electrical equipment, because of its low-dielectric properties, low toxicity, and high stability^1^,^2^,^3^. Despite these advantages, SF_6_ has a global warming potential that is 24000 times larger than that of CO_2_. The restriction of SF_6_ emissions is therefore necessary to allow the use of SF_6_ in applications in a variety of fields^4^,^5^,^6^. The mixing of SF_6_ with N_2_ can reduce SF_6_ emissions, and can save costs in industrial applications. SF_6_-N_2_ mixed gases retain insulating properties, even with low SF_6_ contents (SF_6_:N_2_ = 1:9)^7^,^8^. Mixed SF_6_-N_2_ gases have therefore been widely applied, and the purification and recovery of SF_6_ have become more important. The separation of gases is typically achieved using liquefaction, adsorption, or membrane separation techniques. In liquefaction techniques using refrigeration, it is possible to separate a target molecule by choosing an appropriate temperature near the boiling temperature. However, the separation of SF_6_ via liquefaction is costly, because the system must be maintained at 209 K (the boiling temperature of SF_6_). Liquefaction techniques that use pressurization also have high energy costs; the liquefaction of pure and mixed SF_6_ gases (SF_6_:N_2_ = 1:9) at 293 K was observed above 2 and 20 MPa, respectively^8^,^9^. The hydration of SF_6_ and N_2_ was also conducted under high pressures^10^. Motivated by these high costs, adsorption^8^,^11^,^12^,^13^,^14^,^15^ and membrane separation^9^,^16^,^17^,^18^ have been proposed as low-energy-cost techniques, and the adsorption technique shows promise for applications in a variety of fields.

SF_6_ was preferentially adsorbed over N_2_ in porous media, because of its strong adsorption potential; in contrast, SF_6_ was rarely adsorbed in extremely narrow nanopores^8^,^11^,^12^,^13^,^14^,^15^. SF_6_ adsorption was rarely observed in zeolites with pores with a diameter of 0.5 nm, but N_2_ was adsorbed easily in such narrow nanopores^8^; in contrast, zeolites with pores with a diameter of 1.0 nm selectively adsorbed SF_6_ molecules over N_2_ molecules^11^. Metal organic frameworks, which have uniform and narrow nanopores, also show highly selective adsorption^12^,^13^. The pore size dependence of the SF_6_-and-N_2_ separation abilities of mesoporous silica and zeolite-templated carbons was evaluated by Builes *et al.*, using grand canonical Monte Carlo simulations^14^; 1.1 nm-nanopores separated SF_6_ and N_2_ well. Those preceding studies demonstrated the SF_6_ separation efficiency of various porous media having narrow pores, whereas in pores larger than 2.0 nm, the SF_6_ and N_2_ separation has been neglected. The mechanisms responsible for the adsorption and separation of SF_6_ also have not yet been sufficiently clarified. Thus, narrow and wide pores have been simply considered as having the high separation ability in small pore volumes and low separation ability in large pore volumes, respectively. Porous media having the high separation ability and large pore volumes are ideal materials for application of the separation. We here propose to use wide nanopores for the purpose, because of having relatively high adsorption potentials. In this paper, nanopores are defined as narrower pores than 5 nm.

Nanoporous carbons are composed only of carbon and have simple geometries, and they are therefore useful for examining the above-mentioned adsorption and separation mechanisms. Single walled carbon nanohorns (CNHs) have a tubular structure similar to that of carbon nanotubes^19^,^20^,^21^,^22^. The internal and interstitial sites of CNH particles have cylindrical nanopores with diameters of 2.9 and 0.7 nm, respectively, and the adsorption in the two sets of nanopores can be evaluated separately^21^,^22^. Single-walled carbons also have the potential to adsorb large amounts of SF_6_. Thus, CNHs have the advantages of being a highly efficient separation medium, and allowing the evaluation of the mechanisms responsible for the adsorption and separation of SF_6_. Here, the selective adsorption of SF_6_ over N_2_ in the internal and interstitial nanopores of CNHs was evaluated by measuring the adsorption of SF_6_ and N_2_ at 273 K. The mechanisms responsible for the adsorption and separation of SF_6_ and N_2_ were also investigated in this study.

## Results and Discussion

**Figure 1:**
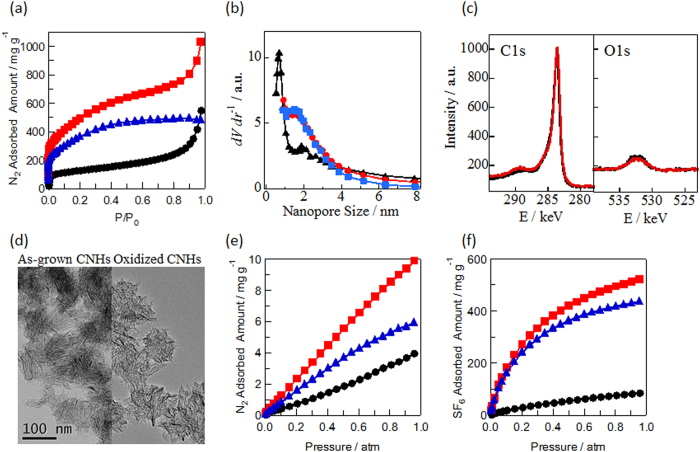
Adsorption isotherms of N_2_ at 77 K on CNHs (**a**) and their pore size distributions (**b**). X-ray photoelectron spectroscopies (**c**) and transmission electron microscopic images (**d**) of as-grown CNHs (black curves) and partially oxidized CNHs (red curves). Adsorption isotherms of N_2_ at 273 K (**e**) and SF_6_ at 273 K (**f**). The symbols represent as-grown CNHs (interstitial nanopores) (●), partially oxidized CNHs (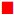), and internal nanopores (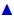).

Both the internal and interstitial nanopores of the partially oxidized CNHs were available for the adsorption of molecules, whereas the internal nanopores of the as-grown CNHs were closed to adsorbed molecules; thus, molecules could be adsorbed in the interstitial nanopores, as reported elsewhere^23^,^24^. The internal nanopores were assessed by measuring the difference between the amounts of N_2_ adsorbed for the partially oxidized CNHs and the as-grown CNHs. The micropore volumes of the interstitial and internal nanopores of the CNHs were determined from the N_2_ adsorption isotherms measured at 77 K on the as-grown and partially oxidized CNHs, as shown in Fig. 1a. The nanopore volumes of the as-grown and partially oxidized CNHs were 0.13 and 0.56 mL g^−1^, respectively, as obtained from the Dubinin-Radushkevich equation^25^. Thus, the nanopore volumes of the interstitial and internal nanopores of the CNHs were 0.13 and 0.43 mL g^−1^, respectively. The adsorption isotherms and porosities of the CNHs agreed with results from previous studies^20^,^21^,^22^,^23^,^24^,^26^. The nanopore size distributions in Fig. 1b were obtained using the Barrett-Joyner-Halenda theory^27^. The interstitial and internal nanopore sizes of the CNHs were distributed mainly in the ranges of <1.2 nm and 1–4 nm, respectively. Thus, the interstitial and internal nanopores were named as narrow and wide nanopores, respectively. The X-ray photoelectron spectroscopies in Fig. 1c and transmission electron microscopic images in Fig. 1d indicated that geometrical and chemical structures were rarely changed by the partial oxidation of as-grown CNHs. The O/C ratios were approximately 5% and those CNHs had less surface oxygen groups. Thus, we discussed the change of selectivity by nanopore size. Figure 1e,f shows N_2_ and SF_6_ adsorption isotherms measured for the CNHs at 273 K. The N_2_ adsorption isotherms measured at 273 K were Henry-type, and the adsorbed amounts were small. In contrast, SF_6_ was adsorbed well in these nanopores even at 1 atm, because of the strong intermolecular interactions of the SF_6_, corresponding to P/P_0_ = 0.07. Because the amounts of SF_6_ adsorbed in the interstitial and internal nanopores were significantly different, the SF_6_ was less densely packed in the interstitial, narrow nanopores with a diameter of 0.7 nm.

**Figure 2:**
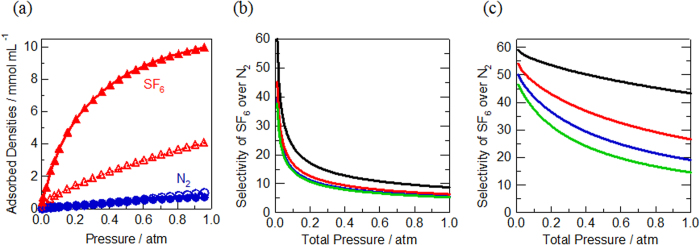
(**a**) Adsorbed densities of N_2_ (●) and SF_6_ (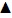) in the interstitial nanopores (open symbols) and internal nanopores of CNHs (filled symbols) at 273 K. Selectivity of SF_6_ over N_2_ in the interstitial (**b**), and internal (**c**) nanopores. The black, red, blue, and green curves represent *y*_*SF6*_:*y*_*N2*_ = 0.1:0.9, 0.3:0.7, 0.5:0.5, and 0.8:0.2, respectively.

The adsorption density results for SF_6_ and N_2_ in the interstitial and internal nanopores (shown in Fig. 2a) clearly showed that the SF_6_ adsorption density was significantly higher in the internal nanopores than in the interstitial nanopores, whereas the N_2_ adsorption density in the interstitial nanopores was higher than in the internal nanopores, as reported elsewhere^28^. Here, the adsorption densities were obtained from the number of adsorbed molecules and the micropore volume. The slightly higher density of N_2_ in the interstitial nanopores was a result of the stronger adsorption potential in the interstitial nanopores, compared with that in the internal nanopores. The significantly lower density of SF_6_ in the interstitial nanopores was a result of the size restriction imposed by the narrow nanopores, which had a diameter of 0.7 nm. Thus, the adsorption density was controlled by two factors: the adsorption potential, and the steric restriction. These factors led to the highly selective adsorption of SF_6_ and N_2_. The selectivity for SF_6_ over N_2_ was defined by a theoretical ideal adsorption expression, as follows^29^:


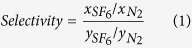


Here, *x*_*i*_ and *y*_*i*_ are the molar fractions of molecule *i* in the adsorbed and bulk phases, respectively. This theoretical ideal adsorption expression has been adopted previously to evaluate the selectivity of binary mixture gas adsorption at relatively low pressures in various porous media^12^,^30^,^31^,^32^,^33^,^34^. The selectivity for the adsorption of SF_6_ over N_2_ in the interstitial and internal nanopores of the CNHs (shown in Fig. 2b,c) was determined from the adsorption densities shown in Fig. 2a. The selectivity is a measure of the molecular sieving ability^8^,^35^,^36^,^37^. The selectivity in the interstitial nanopores decreased exponentially from 60 to 10 at *y*_*SF6*_:*y*_*N2*_ = 0.1:0.9, which is a preferable molar fraction for industrial applications^7^,^8^. For the interstitial nanopores, the selectivity was consistently the greatest at *y*_*SF6*_:*y*_*N2*_ = 0.1:0.9, for all of the molar fractions analyzed in this study. In the internal nanopores, the selectivity was also consistently the greatest at *y*_*SF6*_:*y*_*N2*_ = 0.1:0.9; however, the decreases in the selectivity with increasing pressure were more moderate than those observed in the interstitial nanopores. The exponential decrease of the selectivity observed in the interstitial nanopores was a result of the restriction of the high-density SF_6_ adsorption by the narrow nanopores. At very low pressures, the strong intermolecular potential of SF_6_ promoted the adsorption of SF_6_ in the nanopores, but N_2_ was rarely adsorbed. In the internal nanopores with a diameter of 2.9 nm (i.e., in wide nanopores), SF_6_ was adsorbed without any imposition of a size restriction by the nanopores. Thus, the strong adsorption potential of SF_6_ resulted in high selectivity over the entire range of pressures. High selectivity was therefore achieved via the negative contribution of the steric restriction produced by the narrow interstitial nanopores alone, and the positive contribution of the strong adsorption potential of SF_6_ in both the narrow interstitial and wide internal nanopores. At *y*_*SF6*_:*y*_*N2*_ = 0.1:0.9, the selectivity was 44 in the internal nanopores at 1 atm, which was equivalent to the greatest selectivity value determined in previous studies, as shown in Table 1^11^,^12^,^13^. Thus, the results of this study indicated that the wide nanopores with large pore volumes provided the greatest selectivity—yielding values similar to the greatest values achieved previously in extremely narrow nanopores, under ambient conditions—although grand canonical Monte Carlo simulations suggested that wide nanopores rarely show separation abilities at high pressures^14^.

In this study, the SF_6_ and N_2_ separation abilities of nanoporous carbons were evaluated using adsorption isotherms measured for the narrow interstitial and wide internal nanopores of CNHs (considered as narrow and wide nanopores, respectively). The narrow nanopores of the CNHs had an average diameter of 0.7 nm, and showed high SF_6_ separation abilities in the low-pressure regime; however, the selectivity decreased rapidly with increasing pressure. In contrast, the selectivity in the wide nanopores, which had an average diameter of 2.9 nm, was maintained over the entire range of pressures. These results showed that the wide nanopores functioned well as sites for the separation of SF_6_.

## Methods

CNHs were prepared by the Iijima and Yudasaka groups^19^. The internal nanopores were made accessible using partial oxidation, which was performed at 673 K for 1 h, in an atmosphere of flowing O_2_ gas (using a flow rate of 100 mL min^−1^). N_2_ adsorption isotherms were measured at 77 K and 273 K, and SF_6_ adsorption isotherms were measured at 273 K; in both cases the adsorption isotherms were measured for CNHs and open-CNHs using a volumetric apparatus (Autosorb-1, Quantachrome Co., Florida, USA), after heating at 423 K for more than 2 h, at pressures below 10 mPa. Dubinin-Radushkevich analysis was conducted for the above adsorption isotherms, to evaluate the micropore volumes. The micropore volumes were evaluated using a Dubinin-Radushkevich analysis range of [*ln* (*P*/*P*_*0*_)]^2^ = 20–60, which is typically applied for the analysis of micropores. X-ray photoelectron microscopy (Mg Kα radiation at 10 kV and 10 mA; JPS-9010MX, JEOL Co., Tokyo, Japan) was used to assess the surface oxygen groups in CNHs. Transmission electron microscopy at 120 keV (JEM-2100F, JEOL Co., Tokyo, Japan) was used for direct observations of CNHs.

## Additional Information

**How to cite this article**: Takase, A. *et al.* Wide Carbon Nanopores as Efficient Sites for the Separation of SF6 from N2. *Sci. Rep.* **5**, 11994; doi: 10.1038/srep11994 (2015).

## Figures and Tables

**Table 1:** Experimentally determined literature values for SF_6 _/N_2_ selectivity at approximately 1.0 atm, for mixed gases at a molar fraction of y_SF6_:y_N2_ = 0.1:0.9 in the literature.

Adsorbent	Temperature/ K	Selectivity	Reference
Zn-MOF-74	298	46	Kim *et al.* 2014^12^
Co-MOF-74	298	35	Kim *et al.* 2014^12^
Mg-MOF-74	298	20	Kim *et al.* 2014^12^
MIL-100	298	24	Kim *et al.* 2015^13^
Na-X type zeolite	293	44	Murase *et al.* 2004^11^
Internal nanopores of CNHs	273	44	This study

## References

[b1] Maiss M. & Brenninkmeijer C. A. M. Atomspheric SF_6_: Trends, Sources and Prospects. Environ. Sci. Technol. 32, 3077–3086 (1998).

[b2] Chiang Y. C. & Wu P.-Y. Adsorption equilibrium of sulfur hexafluoride on multi-walled carbon nanotubes. *J. Hazard. Mater*. 178, 729–738 (2010). 20185236 10.1016/j.jhazmat.2010.02.003

[b3] Fang X. K. *et al.* Sulfur Hexafluoride (SF_6_) Emission Estimates for China: An Inventory for 1990–2010 and a Projection to 2020. Environ. Sci. Technol. 47, 3848–3855 (2013). 23506443 10.1021/es304348x

[b4] Ravishankara A. R., Solomon S., Turnipseed A. A. & Warren R. F. Atmospheric Lifetimes of Long-Lived Halogenated Species. Science 259, 194–199 (1993). 17790983 10.1126/science.259.5092.194

[b5] Mohindra V., Chase H., Sawin, H. H. & Mocella M. T. Abatement of perfluorocom-pounds (PFCs) in a microwave tubular reactor using O_2_ as an additive gas. IEEE Trans. Plasma Sci. 10, 399–411 (1997).

[b6] Langan J., Maroulis P. & Ridgeway R. Strategies for greenhouse gas reduction. Solid State Technol. 39, 115–121 (1996).

[b7] Inami K. *et al.* Problems of the Application of N_2_/SF_6_ Mixtures to Gas-Insulated Bus. Electr. Eng. Jpn. 137, 25–31 (2001).

[b8] Toyoda M. *et al.* SF_6_ Reclaimer From SF_6_/N_2_ Mixtures by Gas Separation With Molecular Sieving Effect. IEEE Trans. Power. Deliv. 18, 442–448 (2003).

[b9] Yamamoto O., Takuma T. & Kinouchi M. Recovery of SF_6_ from N_2_/SF_6_ gas mixtures by using a polymer membrane. IEEE Electr. Insul. Mag. 18, 32–37 (2002).

[b10] Cha I., Lee S., Lee J. D., Lee G.-W. & Seo Y. Separation of SF_6_ from Gas Mixtures using Gas Hydrate Formation. Environ. Sci. Technol. 44, 6117–6122 (2010). 20704207 10.1021/es1004818

[b11] Murase H., Imai T., Inohara T. & Toyoda M. Use of Zeolite Filter in Portable Equipment for Recovering SF_6_ in SF_6_/N_2_ Mixtures. IEEE Trans. Electr. Insul. 11, 166–173 (2004).

[b12] Kim M. B., Lee S. J., Lee C. Y. & Bae Y. S. High SF_6_ selectivities and capacities in isostructural metal-organic frameworks with proper pore sizes and highly dense unsaturated metal sites. Micropor. Mesopor. Mat. 190, 356–361 (2014).

[b13] Kim P.–J. *et al.* Separation of SF_6_ from SF_6_/N_2_ mixture using metal-organic framework MIL-100(Fe) granule. Chem. Eng. J. 262, 683–690 (2015).

[b14] Builes S., Roussel T. & Vega L. F. Optimization of the Separation of Sulfur Hexafluoride and Nitrogen by Selective Adsorption Using Monte Carlo Simulations. AIChE J. 57, 962–974 (2011).

[b15] Cho W. S., Lee K. H., Chang H. J., Huh W. & Kwon H. H. Evaluation of pressure-temperature swing adsorption for sulfur hexafluoride (SF_6_) recovery from SF_6_ and N_2_ gas mixture. Korean J. Chem. Eng. 28, 2196–2201 (2011).

[b16] Shiojiri K., Yanagisawa Y., Yamasaki A. & Kiyono F. Separation of F-gases (HFC-134a and SF_6_) from gaseous mixtures with nitrogen by surface diffusion through a porous Vycor glass membrane. J. Membr. Sci. 282, 442–449 (2006).

[b17] Shao L., Low B. T., Chung T. S. & Greenberg A. R. Polymeric membranes for the hydrogen economy: Contemporary approaches and prospects for the future. J. Membr. Sci. 327, 18–31 (2009).

[b18] Kim D. H., Ko Y. H., Kim T. H., Park J. S. & Lee H.-K. Separation of N_2_/SF_6_ binary mixtures using polyethersulfone (PESf) hollow fiber membrane. Korean J. Chem. Eng. 29, 1081–1085 (2012).

[b19] Iijima S. *et al.* Nano-aggregates of single-walled graphitic carbon nano-horns. Chem. Phys. Lett. 309, 165–170 (1999).

[b20] Murata K. *et al.* Pore structure of single-wall carbon nanohorn aggregates. Chem. Phys. Lett. 331, 14–20 (2000).

[b21] Ohba T. *et al.* N_2_ Adsorption in an Internal Nanopore Space of Single-Walled Carbon Nanohorn: GCMC Simulation and Experiment. Nano Lett. 1, 371–373 (2001).

[b22] Ohba T. *et al.* Quasi One-Dimensional Nanopores in Single-Wall Carbon Nanohorn Colloids Using Grand Canonical Monte Carlo Simulation Aided Adsorption Technique. J. Phys. Chem. B 109, 8659–8662 (2005). 16852025 10.1021/jp0503011

[b23] Murata K. *et al.* Nanowindow-Induced Molecular Sieving Effect in a Single-Wall Carbon Nanohorn. J. Phys. Chem. B 106, 12668–12669 (2002).

[b24] Utsumi S. *et al.* Opening Mechanism of Internal Nanoporosity of Single-Wall Carbon Nanohorn. J. Phys. Chem. B 105, 14319–14324 (2005). 16852800 10.1021/jp0512661

[b25] Dubinin M. M. The Potential Theory of Adsorption of Gases and Vapors for Adsorbents with Energetically Nonuniform Surfaces. Chem. Rev. 60, 235–241 (1960).

[b26] Ohba T., Yamamoto S., Takase A., Yudasaka M., & Iijima S. Evaluation of carbon nanopores using large molecular probes in grand canonical Monte Carlo simulations and experiments. Carbon 88, 133–138 (2015).

[b27] Barrett E. P., Joyner L. G. & Halenda P. P. The determination of pore volume and area distributions in porous substances. I. Computations from nitrogen isotherms. J. Am. Chem. Soc. 73, 3155–3158 (1951).

[b28] Ohba T. The thinnest molecular separation sheet by graphene gates of single-walled carbon nanohorns. ACS Nano 8, 11313–11319 (2014). 25347389 10.1021/nn504162s

[b29] Myers A. L. & Prausnitz J. M. Thermodynamics of Mixed-Gas Adsorption. AIChE J. 11, 121–127 (1964).

[b30] Cannon J. J., Vlugt T. J. H., Dubbeldam D., Maruyama S. & Shiomi J. Simulation Study on the Adsorption Properties of Linear Alkanes on Closed Nanotube Bundles. J. Phys. Chem. B 116, 9812–9819 (2012). 22764885 10.1021/jp3039225

[b31] Jakobtorweihen S. & Keil F. J. Adsorption of alkanes, alkenes and their mixtures in single-walled carbon nanotubes and bundles. Mol. Simulat. 35, 90–99 (2009).

[b32] Furmaniak S. *et al.* Surface to volume ratio of carbon nanohorn – A crucial factor in CO_2_/CH_4_ mixture separation. Chem. Phys. Lett. 595, 67–72 (2014).

[b33] Bae Y. S., Farfa O. K., Hupp J. T. & Snurr R. Q. Enhancement of CO_2_/N_2_ selectivity in a metal-organic framework by cavity modification. J. Mater. Chem. 19, 2131–2134 (2009).

[b34] Babarao R., Hu Q. Z., Jiang J. W., Chempath S. & Sandler I. S. Diffusion and Separatoin of CO_2_ and CH_4_ in Silicalite, C_168_ Schwarzite, and IRMOF-1: A Comparative Study from Molecular Simulation. Langmuir 24, 5474–5484 (2008). 18433152 10.1021/la703434s

[b35] Ohba T., Kanoh H. & Kaneko K. Superuniform Molecular Nanogate Fabrication on Graphene Sheets of Single Wall Carbon Nanohorns for Selective Molecular Separation of CO_2_ and CH_4_. Chem. Lett. 40, 1089–1091 (2011).

[b36] Lopez-Ramon M. V., Jagiello J., Bandosz T. J. & Seaton N. A. Determination of the Pore Size Distribution and Network Connectivity in Microporous Solids by Adsorption Measurements and Monte Carlo Simulation. Langmuir 13, 4435–4445 (1997).

[b37] Cao D. V. & Sircar S. Heat of Adsorption of Pure Sulfur Hexafluoride on Micro-Mesoporous Adsorbents. Adsorption 7, 73–80 (2001).

